# Granular Cell Tumours: A Rare Entity in the Musculoskeletal System

**DOI:** 10.1155/2009/765927

**Published:** 2010-02-07

**Authors:** Barry Rose, George S. Tamvakopoulos, Eric Yeung, Robin Pollock, John Skinner, Timothy Briggs, Steven Cannon

**Affiliations:** London Bone and Soft-Tissue Tumour Unit, Royal National Orthopaedic Hospital NHS Trust, Brockley Hill, Stanmore, Middlesex HA7 4LP, UK

## Abstract

Granular Cell Tumours are rare mesenchymal soft tissue tumours that arise throughout the body and are believed to be of neural origin. They often present as asymptomatic, slow-growing, benign, solitary lesions but may be multifocal. 1-2% of cases are malignant and can metastasise. Described series in the literature are sparse. We identified eleven cases in ten patients treated surgically and followed-up for a period of over 6 years in our regional bone and soft tissue tumour centre. Five tumours were located in the lower limb, four in the upper limb, and two in the trunk. Mean patient age was 31.2 years (range 8–55 years). Excision was complete in one case, marginal in five cases and intralesional in five cases. No patients required postoperative adjuvant treatment. Mean follow-up was 19.3 months (range 1–37 months). One case was multifocal, but there were no cases of local recurrence or malignancy. Histopathological and immunohistochemical analysis revealed the classical granular cell tumour features in all cases. We believe this case series to be the largest of its type in patients presenting to an orthopaedic soft tissue tumour unit. We present our findings and correlate them with findings of other series in the literature.

## 1. Introduction

Granular cell tumour was originally described in the German literature as granular cell myoblastoma in 1926 [1]. Current opinion considers the tumour to be neural in origin [2, 3].

Clinically, these tumours are rare and account for approximately 0.5% of all soft tissue tumours [4]. Most of the published experience arises from sparsely presented case reports and few small series [4–7]. For the most part, granular cell tumours behave in a benign fashion, but have a tendency to recur. They can be multifocal at presentation. They can arise anywhere in the body, typically in the superficial tissues (dermis and subcutis), along mucosal surfaces and occasionally within skeletal muscle. In rare cases they can metastasise, particularly when they arise in deep to fascia or are over 4 cm in diameter [7]. Malignant transformation is rare but well recognised.

## 2. Materials and Methods

A retrospective review of our database was carried out. All patients with a diagnosis of granular cell tumour were included in the study. Our study covered the period 2002–2008. All patients underwent preoperative MRI scanning. Patients were treated with either core needle biopsy followed by wide local excision or by excision biopsy.

## 3. Results

Results are presented in Table 1. Eleven tumours were identified in ten patients, all located within the soft tissues. There was one male patient and nine female patients. The mean age was 31.2 years (range 8–55 years). Five tumours were located in the lower limb (three thigh, two calf), four in the upper limb (two arm, one forearm, one finger), and two within the trunk (one thoracic spine musculature, one supraclavicular fossa). Six of the resected lesions were subcutaneous (54.5%) and five intramuscular (45.5%). The history of symptom duration was available in nine out of eleven patients, with a mean of 10.3 months (range 3–24 months).

Seven cases had a preoperative core needle biopsy (63.3%), and four underwent excision biopsy. All tumours were treated with surgical excision. Eighteen resection and biopsy specimens were therefore examined.

The core needle biopsy was successful in diagnosing granular cell tumour in all cases. Excision was wide (7 mm margin) in one resection case (9.1%), marginal in five cases (45.5%), and intralesional in the remaining five cases (45.5%). Marginally excised cases all had a 1mm margin of normal tissue. The mean diameter of the tumours was 38.4 mm (range 10–130 mm).

**Figure 1:**
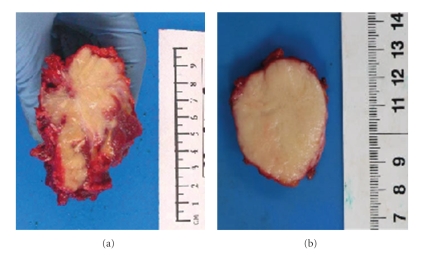
Gross appearance of granular cell tumour. This is an example of multicentric granular cell tumour (case number 10). On the left side (a), the tumour is poorly circumscribed solid and pale yellow, and shows infiltrative growth margin (paraspinal tumour). On the right side (b) the tumour shows better defined pushing margin (supraclavicular tumour).

**Figure 2:**
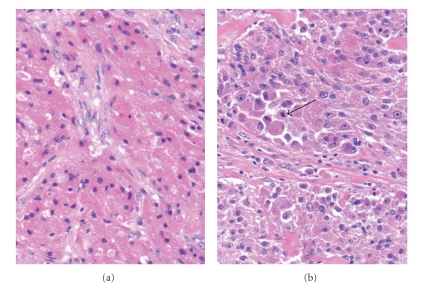
Typical histological appearance of granular cell tumour (a). Sheets of rounded and oval cells with large amount of fine granular eosinophilic cytoplasm. The nuclei are bland and eccentric (20×, H&E stain). Atypical granular cell tumour (b). In comparison with Figure 2(a), there is nuclear pleomorphism, prominent eosinophilic nucleoli, and cell spindling. A mitosis is also present (arrow) (20×, H&E stain).

In all cases histopathological examination showed the classical features of granular cell tumour, the hallmark of which is the finding of large cells containing a finely granular cytoplasm (Figures 1 and 2). All specimens were benign, with two (diameters 40 mm and 42 mm) displaying pathological features of atypia (necrosis, vesicular nuclei with large nucleoli, pleomorphism, increased mitotic rate). As described by Fanburg-Smith et al., six histologic criteria are important in defining malignancy in granular cell tumours: necrosis, cell spindling, vesicular nuclei with large nucleoli, increased mitotic rate (>2/10 high-power fields), high nuclear-to-cytoplasmic ratio and pleomorphism. Three or more criteria present define malignancy, one or two define atypia, and none or solely pleomorphism define benign tumours [7]. Sixteen out of seventeen (94.1%) specimens showed no necrotic features, with one specimen (5.9%) displaying elements of necrosis. Prominent nucleoli were seen in six out of seventeen specimens (35.3%), and nuclear pleomorphism in two out of seventeen specimens (11.8%). The mitotic rate ranged from 0 to 2/10 high-power fields, which was highest in the two atypical tumours. Typically, tumour cells were diffusely and strongly positive for S100 protein by immunohistochemistry; whereas the other markers tested were negative. These included smooth muscle actin, desmin, myogenin, and pankeratin MNF116.

No patients received chemotherapy or radiotherapy at any point. One patient was lost to follow-up following surgery. Mean follow-up of the remaining cases was 19.3 months following surgical excision (range 1–55 months). There were no cases of local recurrence. One patient experienced distant recurrence, representing with a supraclavicular fossa tumour following intralesional excision of a thoracic spine tumour. One patient developed Chronic Regional Pain Syndrome post-operatively, which recovered following conservative management.

## 4. Discussion

Granular cell tumours are rare and the literature relating to them is sparse. Abrikossoff was the first to describe this tumour in the literature in 1926 [1] as a myoblastoma, since it was reported as a tumour arising from muscle in the tongue. Over the following 60 years the tumour was thought to be neural in origin since it was observed that tumours arising in the extremities were related to the radial and sciatic nerve trunks [3, 8, 9]. Current opinion concurs with this.

Lack et al. in 1980 [2] were the first to scrutinise the collection of histopathological specimens labelled as granular cell tumours in two centres and correlate them with clinical presentation [2]. They reported on a total of 118 cases of granular cell tumours in all localities. The most common site was the tongue although greater numbers of tumours seemed to occur in the skin and subcutaneous tissues. 

Tsuchida et al. reviewed the reported cases of granular cell tumours in the English and Japanese literature [4]. They found a 2.9 : 1 female to male preponderance for the tumour and compared it with previous reported series (1.8:1 and average age 32 years [2]). 26.8% of cases were in the lower extremities and previous reports showed that benign tumours were uncommon (6.4%) in the lower extremities. Of 11 lesions presenting in the upper and lower extremities 36% were within muscle and only 4% of those were benign. 

The mode of presentation of granular cell tumours is usually in the form of a painless mass and occurs most commonly between the third and fifth decades [2, 4, 7]. The mean age in our series was 31.2 years. They occur more commonly in women than in men and have preponderance for patients of Afro-Caribbean race. The reported ratio of female to male cases is 1.8–2.9 : 1 [2, 4, 7] although in our series the ratio was much higher at 9:1.

One feature of these tumours is that they may be multicentric or multifocal at time of presentation. This raises the suspicion of malignancy. The reported incidence of multicentricity at time of presentation ranges between 5 and 16%. In our series we had one patient with two separate lesions, one on the dorsal chest wall and another in the supraclavicular fossa. Both lesions were benign. In our opinion this represents multifocal rather than metastatic disease.

Fanburg-Smith et al. [7] defined the diagnostic criteria for malignant granular cell tumours and correlated their findings with previous series. They reported 73 cases, 28 of which were malignant, 3 cases were multicentric and 9 cases were atypical. They found that the multicentric and atypical cases all ran a benign course exhibiting no evidence of metastasis and no deaths. They did, however, observe in one multicentric case that a new tumour arose in a new location. In the atypical group there were local recurrences but no deaths after 8 years of follow-up. In 28 malignant cases they had a 39% death rate with a median interval of 3 years compared with 50% at 2.8 years in the Tsuchida series [4]. They reported a rate of metastasis of 50%, with lymph nodes and bone being the most common sites. Interestingly the local recurrence rate of patients with metastasis was 32% (compared with 50% in Tsuchida's series).

The best radiological modality for characterisation is Magnetic Resonance Imaging (MRI) [8]. Benign granular cell tumours are classically iso-intense or brighter than muscle on T1-weighted sequences, round or oval in shape, superficial in location, and 4 cm or less in size. On T2-weighted sequences, the signal from the central portion of the lesion is classically iso-intense to muscle or suppressed fat, with high signal peripherally. Malignant granular cell tumours may demonstrate invasion of adjacent structures and signal intensity characteristics that are often seen with other aggressive neoplasms.

Histopathological examination classically reveals large cells with an eosinophilic granular cytoplasm, as was demonstrated in all specimens in our series. All granular cell tumours tested S-100 positive [7]. The lack of melanosomes and negative HMB45 immunostaining in granular cell tumours help distinguish it from malignant melanoma. The schwannian features of malignant peripheral nerve sheath tumours (MPNST) and lack of S-100 staining help distinguish them from granular cell tumours and therefore enable exclusion from the differential diagnosis.

Malignant granular cell tumours comprise 1%-2% of all granular cell tumours. They occur most commonly at 40–50 years of age [4, 7], and in female patients. Tsuchida et al. [4] report 26 out of 35 (74%) and Fanburg-Smith et al. [7] report 32 out of 46 patients (70%). Risk of malignancy is increased if tumour size is greater than 4 cm at presentation [7]. 91% of the resected tumours in our series had a longest diameter of less than 42 mm. Although the largest two of these specimens had atypical features, no specimens in our series were malignant. Interestingly, by far the largest tumour resected did not show any features of atypia or malignancy, despite this being a distant recurrence. In our series, the depth of the tumour, and tissue within which it arose had no correlation with the risk of malignancy. The margin of resection in our series bore no correlation with the risk of malignancy or recurrence. It would be logical to hypothesise that intralesional excision would give rise to an increased risk of local recurrence or metastasis, but this is not a trend that we identified in our series.

In our series we had 100% patient survival rate at a mean of 19.3 months post-operatively (range 1–55 months) on a background of symptoms of mean duration 10.4 months (range 3–24). Survivorship has been described as worsening with size greater than 4 cm, female gender, tumour presenting in the lower extremities, intramuscular location, rapid recent growth, local recurrence, metastases, Ki67 values greater than 10% and p53 immunoreactivity [7]. Radiotherapy and chemotherapy have not been shown to significantly improve the clinical course of the disease [10].

We present a series of eleven cases of granular cell tumours in ten patients, with one case of distant recurrence. All tumours were benign, although the largest two primary tumours did display atypical histopathological features. In our series, the depth of the tumour, the tissue in which it was located, the size and the margins of resection all bore no correlation with the risk of malignancy or short-term survivorship. Medium-term and long-term follow-up is required to elucidate this further.

## Figures and Tables

**Table 1:** Patient data and histological features^a^.

Patient	Age	Tumour location	Size in mm (widest diameter)	Depth	Resection Margin (mm)	Atypia	Malignancy
1	39	Thigh	20	SC	1	N	N
2	11	Calf	40	IM	Intralesional	N	N
3	30	Forearm	20	SC	Intralesional	N	N
4	37	Arm	U	SC	1	N	N
5	37	Calf	22	IM	1	N	N
6	29	Deltoid	35	IM	1	N	N
7	40	Thigh	42	SC	Intralesional	Y	N
8	55	Thigh	20	SC	7	N	N
9	8	Finger	10	SC	Intralesional	N	N
10a	26	Posterior thoracic spine musculature	130	IM	Intralesional	N	N
10b	26	Supraclavicular	40	IM	1	Y	N

U: unrecorded; SC: subcutaneous; IM: intramuscular; Y: Yes; N: No.

^a^patient 10 presented with distant recurrence.
